# Surgical Technique of Laparoscopic Whole-Layer Cholecystectomy for Suspected Early Gallbladder Cancer: A Safe and Minimally Invasive Approach

**DOI:** 10.7759/cureus.82873

**Published:** 2025-04-23

**Authors:** Tomokazu Tanaka, Takao Ide, Kotaro Ito, Keita Kai, Hirokazu Noshiro

**Affiliations:** 1 Surgery, Saga University, Saga, JPN; 2 Pathology, Saga University, Saga, JPN

**Keywords:** cystic plate, early-gallbladder cancer, laennec’s capsule, laparoscopic whole layer cholecystectomy, less invasiveness, oncological safety

## Abstract

It is difficult to diagnose early gallbladder cancer (GBC) preoperatively; therefore, the surgical procedure for patients suspected of having this condition should be chosen with curability and invasiveness in mind. We herein present a standardized procedure for performing complete laparoscopic whole-layer cholecystectomy (LWLC) for suspected early GBC.

Between January 2010 and January 2023, LWLC was performed on 44 patients with suspected early GBCs located on the peritoneal side. We devised and performed the following procedure to perform LWLC safely. Our procedure is based on the preservation of Laennec’s capsule with an appropriate transection plane using the cystic plate as an anatomical landmark. The steps of this procedure comprise (1) isolation of the cystic duct and artery with accurate dissection of the 12c and cystic duct lymph nodes and preservation of the hilar plate; (2) preemptive ligation of the cystic duct to seal off any free cancer cells floating in bile within the gallbladder; (3) excision of the entire gallbladder starting from the S4-side of the fundus to neck with preservation of Laennec’s capsule; (4) definitive visual identification and transection of the cystic plate at the neck; and (5) resection of the cystic duct after placement of the gallbladder in an organ bag to prevent intraperitoneal dissemination of bile.

Pathological examination revealed 15 GBCs (34.1%), of which 13 cases were early stage (86.7%). Specifically, the depth of invasion was in situ in four cases, mucosa in four cases, muscle propria in five cases, and subserosa in two cases. Regarding lymph node metastasis, it was detected in only one case, which had tumor invasion of the subserosa. The median operation time was 138 minutes, and estimated blood loss was 27 mL (range: 0-108 mL). There was no intraoperative bile leakage and no postoperative complications requiring surgical, endoscopic, or radiological intervention. We confirmed elastic fibers covering the outer layer of subserosa at the level of dissection from the liver bed. With a median follow-up of 56 months, 14 patients remain alive without recurrence, and one has died from another disease.

Our original procedure of LWLC is a reproducible and feasible means of treating early or suspected GBC located on the free peritoneal side, achieving lengthy remission, being less invasive than other currently performed procedures, and possibly being curative.

## Introduction

Performing laparoscopic surgery for suspected gallbladder cancer (GBC) remains controversial and is not a standard approach. The strongest disadvantages are associated with handling of the tumor during laparoscopic surgery and the potential for peritoneal dissemination and port-site recurrence as a result of intraoperative gallbladder perforation [1,2]. However, it is difficult to diagnose early GBC preoperatively because (1) it is difficult to obtain a sample for preoperative pathological assessment because of its location; (2) coexisting stones and inflammatory changes can obscure imaging findings; and (3) diffuse thickness of the wall can mimic cancer [3,4]. When laparoscopic cholecystectomy, combined with resection of the liver or extrahepatic bile duct or not, is performed for suspected GBC, and the postoperative pathological diagnosis is a benign disease, the surgery performed was overly invasive.

Despite the obstacles to laparoscopic surgery for GBC, laparoscopic cholecystectomy is a standard procedure for benign gallbladder disease. Some studies have reported that laparoscopic cholecystectomy or laparoscopic whole-layer cholecystectomy (LWLC) for early-stage GBC not invading beyond the muscularis propria has the advantages of being minimally invasive and achieving favorable oncological outcomes [5,6]. However, with a laparoscopic approach, there are still concerns about the exposure and spread of cancer cells in bile, necessitating the development of surgical procedures that minimize these concerns.

Thus, the development of treatment strategies and surgical procedures that provide both oncological safety and minimal invasiveness is required for suspected GBC. In the present study, we present the standardized surgical techniques that we have established for LWLC, the landmarks of which include the hilar plate [7], cystic plate, and Laennec’s capsule [8]. The procedure comprises five steps and achieves both oncological safety and minimal invasiveness. Here, we present the short-term surgical results and oncological outcomes.

## Technical report

Materials and methods

Patients

We retrospectively analyzed relevant data of 44 consecutive patients who underwent LWLC for suspected early GBC in the Department of Surgery, Saga University Hospital, Saga, Japan, between January 2010 and January 2023. Suspected early GBC was diagnosed on the basis of findings of preoperative imaging, including abdominal ultrasonography, computed tomography, and magnetic resonance imaging. Depending on the findings of those forms of imaging, fluorodeoxyglucose-positron emission tomography, endoscopic ultrasound, and endoscopic retrograde cholangiopancreatography were also performed. Preoperative imaging findings that aroused suspicion of early GBC comprised Ip or Isp type lesion > 10 mm in diameter [9], increase in tumor size on sequential imaging, sessile lesion [9], inability to rule out malignancy of an irregular wall thickness lesion [10], elevated lesion with dense contrast enhancement [11], and positive accumulation on fluorodeoxyglucose-positron emission tomography [12]. The only selection criterion for LWLC is that the gallbladder lesion was located on the peritoneal side: patients whose gallbladder lesions were located on the liver bed side and/or cystic duct were excluded. This study was reviewed and approved by the Ethics Committee of the Saga University Hospital on October 10th, 2023 (approval number: 2019-09-R-03).

Surgical Procedure

We present the five steps and procedural key points of this surgery in Video 1.

**Video 1:** Surgical procedures of laparoscopic whole-layer cholecystectomy.

**Figure 1:**
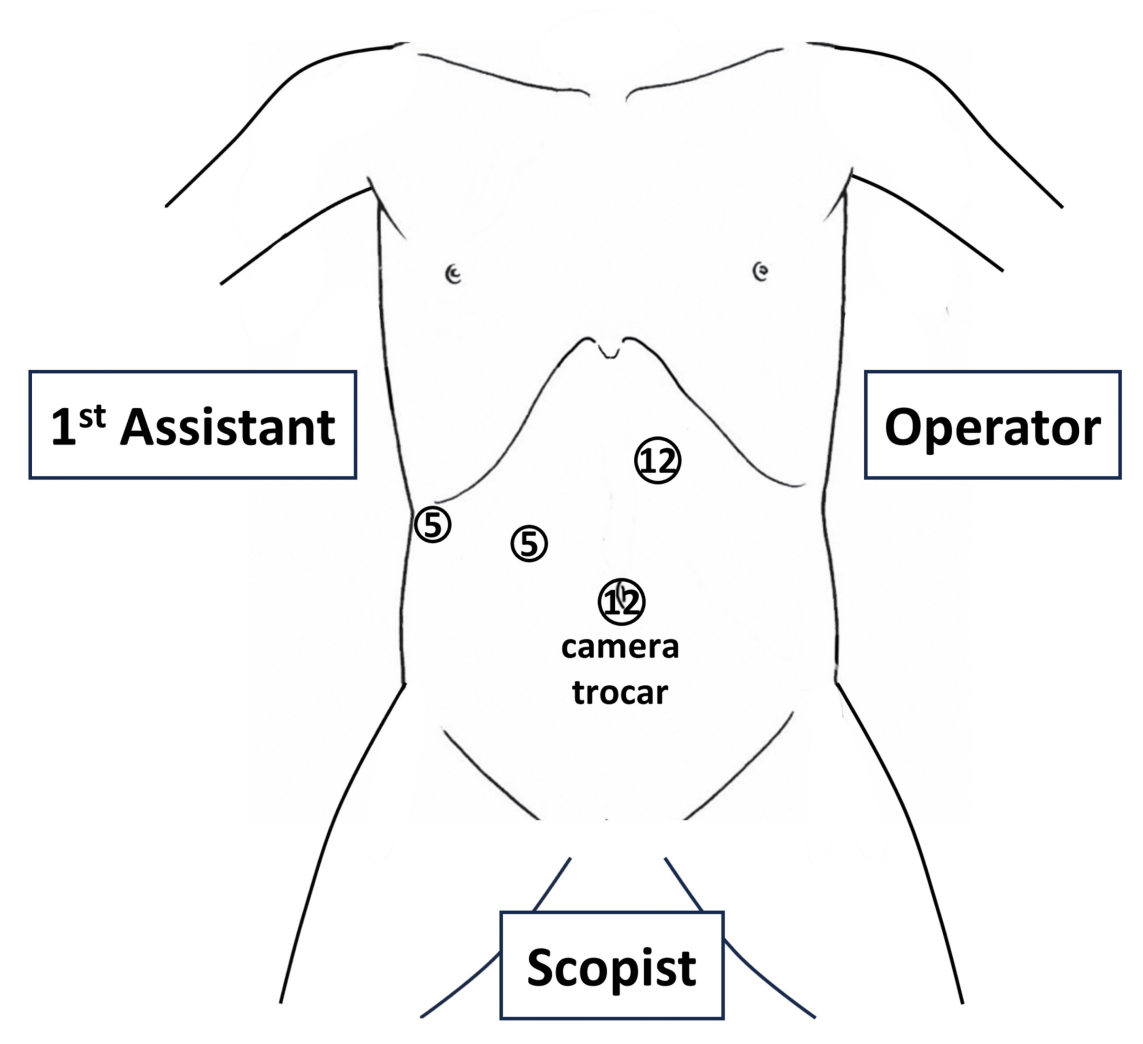
The location of the trocars and the position of surgeons. Source: The figure was purchased from https://note.com/yasuke8499/n/nc54203212f8d, and used under a royalty-free license with permission for inclusion in a published article.

The patient was positioned in an open-leg, head- and right side-up position with the operator standing on their left. First, four trocars (two 12-mm and two 5-mm) were inserted, as shown in Figure 1. A 12-mm flexible camera trocar was then placed at the umbilicus. A pneumoperitoneum was induced and maintained at 10 mmHg.

Before beginning intraabdominal manipulation, intraoperative ultrasonography was implemented to evaluate the location of the gallbladder lesion and confirm the anatomical landmarks of the base of Segment 4 and the roof of Rouvière’s sulcus to minimize injury to the right anterior and posterior Glisson during the surgical procedure. In addition, close attention was paid to identifying intraoperative bile leakage. Furthermore, the operative field was created with folded gauze to protect against gallbladder perforation and minimize manipulation of cancer cells. Isolation of the cystic duct and cystic artery at the gallbladder neck while preserving the hilar plate [7], dissection of the intact gallbladder from the liver bed with preservation of Laennec’s capsule, and transection of the cystic plate - which is continuous with the right anterior Glissonean pedicle [8] - at the gallbladder neck are all important steps that can aid in performing LWLC safely and securely.

Based on these principles, we have developed an original surgical approach consisting of the following five steps: (1) isolation of the cystic duct and cystic artery with preservation of the hilar plate; (2) preemptive transection of the cystic artery and ligation of the cystic duct; (3) complete excision of the intact gallbladder with preservation Laennec’s capsule; (4) visual identification and transection of the cystic plate; and (5) resection of the cystic duct after placement of the gallbladder in an organ bag. Once the resected gallbladder had been completely divided, we generally subjected the stump of the cystic duct to intraoperative frozen section diagnosis. We did not routinely insert a drain.

**Figure 2:**
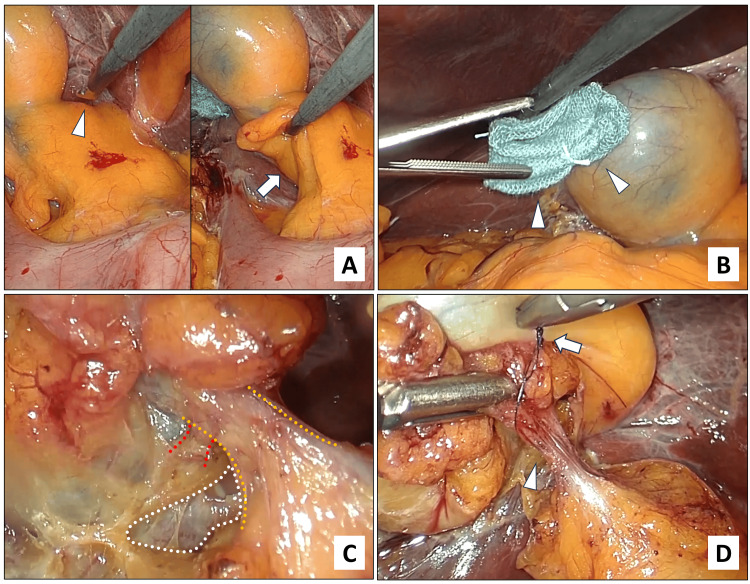
Intraoperative photographs showing management of the cystic duct and artery with preservation of the hilar plate. A. Identification of the base of Segment 4 (white arrowhead) and the roof of Rouvière’s sulcus (white arrow). B. Folded gauze for delineating the operative field and preventing gallbladder perforation (white arrowhead). C. Isolation of the cystic duct (area encircled with yellow dotted lines) and cystic artery (red encircled with white dotted line) with preservation of the hilar plate (area encircled with white dotted line). D. Preemptive ligation of the cystic duct with 4-0 PDS (polydioxanone suture) (white arrow).

**Figure 3:**
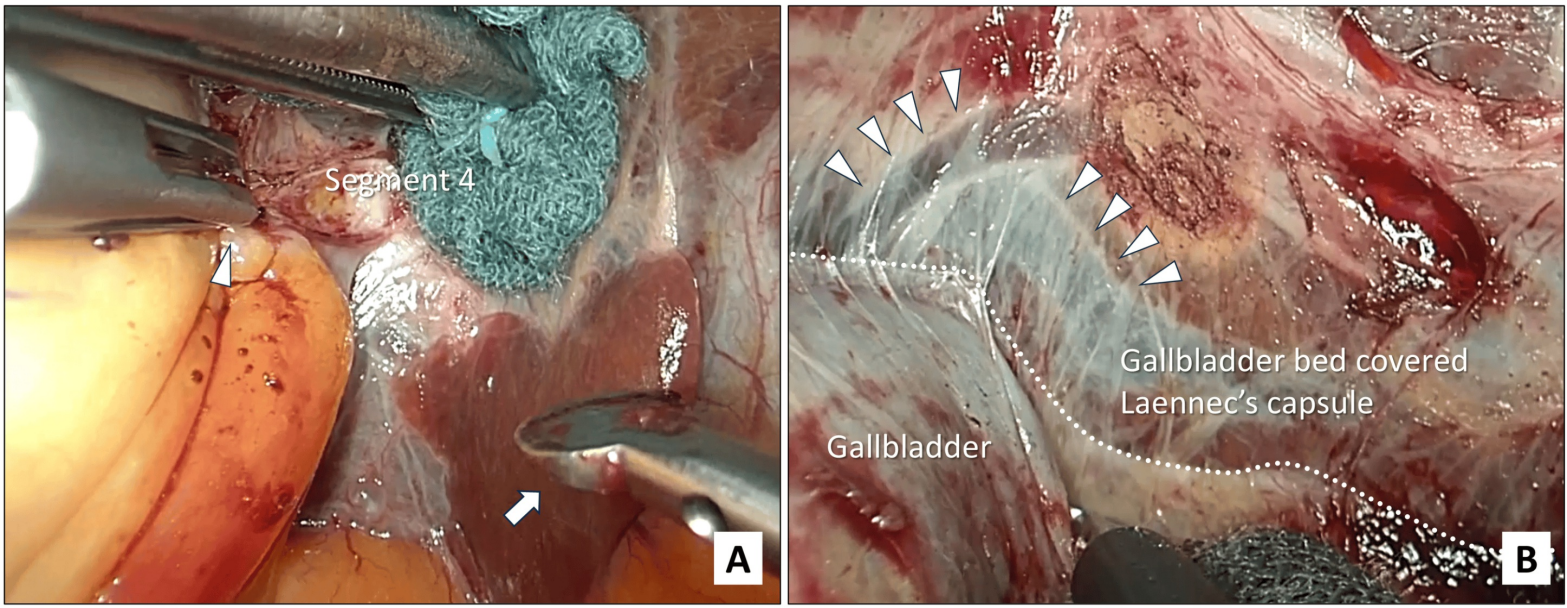
Intraoperative photographs showing whole-layer dissection of the gallbladder while preserving Laennec’s capsule. A. Dissection of the entire gallbladder starting from the S4 side of the fundus and advancing to the neck using fine-tipped forceps (white arrowhead) and an Innovative Operating (IO)-advance electrode capable (AMCO, Tokyo, Japan) (white arrow). B. Whole-layer cholecystectomy is performed while a glossy layer is maintained on the surfaces of both the liver bed and gallbladder sides. Their boundaries are indicated by white dotted lines. The branches of the middle hepatic vein (white arrowheads) can be seen through the Laennec’s capsule.

Here, we detail each step of the procedure:

(1) Isolation of the cystic duct and cystic artery while the hilar plate is preserved: After determining the locations of the base of Segment 4 and the roof of Rouvière’s sulcus (Figure 2A), an assistant creates the operative field by pushing the base of Segment 4 or the gallbladder toward the cranial side, using folded gauze to prevent gallbladder perforation (Figure 2B). Dissection then begins on the right side of the hepato‑duodenal ligament and advances toward Calot's triangle in a line designed to ensure sampling of the 12c and cystic duct lymph nodes. The cystic artery and duct are isolated completely in Calot's triangle, making a critical contribution to safety. In the procedures at the gallbladder neck, dissection must be performed so as to preserve the hilar plate, the goal being to achieve complete excision of the intact gallbladder and fundus (Figure 2C). It is also important to note that the first step serves as a staging laparoscopy, enabling evaluation of the presence or absence of factors that indicate cure is not possible, such as peritoneal dissemination.

(2) Preemptive transection of the cystic artery and ligation of the cystic duct: The cystic artery is dealt with first, followed by the cystic duct. The cystic artery is transected with laparoscopic coagulating shears to reduce bleeding during the removal of the gallbladder from the liver bed. The cystic duct is then ligated using monofilament thread to seal off cancer cells floating in bile within the gallbladder (Figure 2D).

(3) Dissection of the intact gallbladder with preservation of Laennec’s capsule: Dissection of the intact gallbladder starts from the S4 side of the fundus and advances to the neck (fundus first technique) (Figure 3A). It is essential to grasp the gallbladder with fine-tipped forceps such as precision forceps, and to pull the gallbladder while grasping the area near the point of dissection from the liver bed (Figure 3A). The most important goal in this step is to achieve complete dissection of the intact gallbladder while preserving the serosal surface of the gallbladder and Laennec’s capsule (Figure 3B). Identification of the correct plane along which to separate requires constant attention to establishing and maintaining well-balanced countertraction between the gallbladder and liver bed. Dissection in the correct plane reveals a glossy layer on both the gallbladder and the liver bed sides. In addition, bleeding from the gallbladder and liver bed during dissection is minimized, and it is possible to see through the peripheral branches of the middle hepatic vein on the liver bed side. The use of an Innovative Operating (IO)-advance electrode capable of soft coagulation (AMCO, Tokyo, Japan) during complete excision of the intact gallbladder facilitates the maintenance of the correct layers of dissection with hemostasis.

The dissection layer of “laparoscopic whole-layer cholecystectomy” has a higher bleeding risk than that of “laparoscopic simple cholecystectomy” for benign disease. Therefore, this device, which can perform dissection, incision, and soft coagulation, is suitable for our procedures.

**Figure 4:**
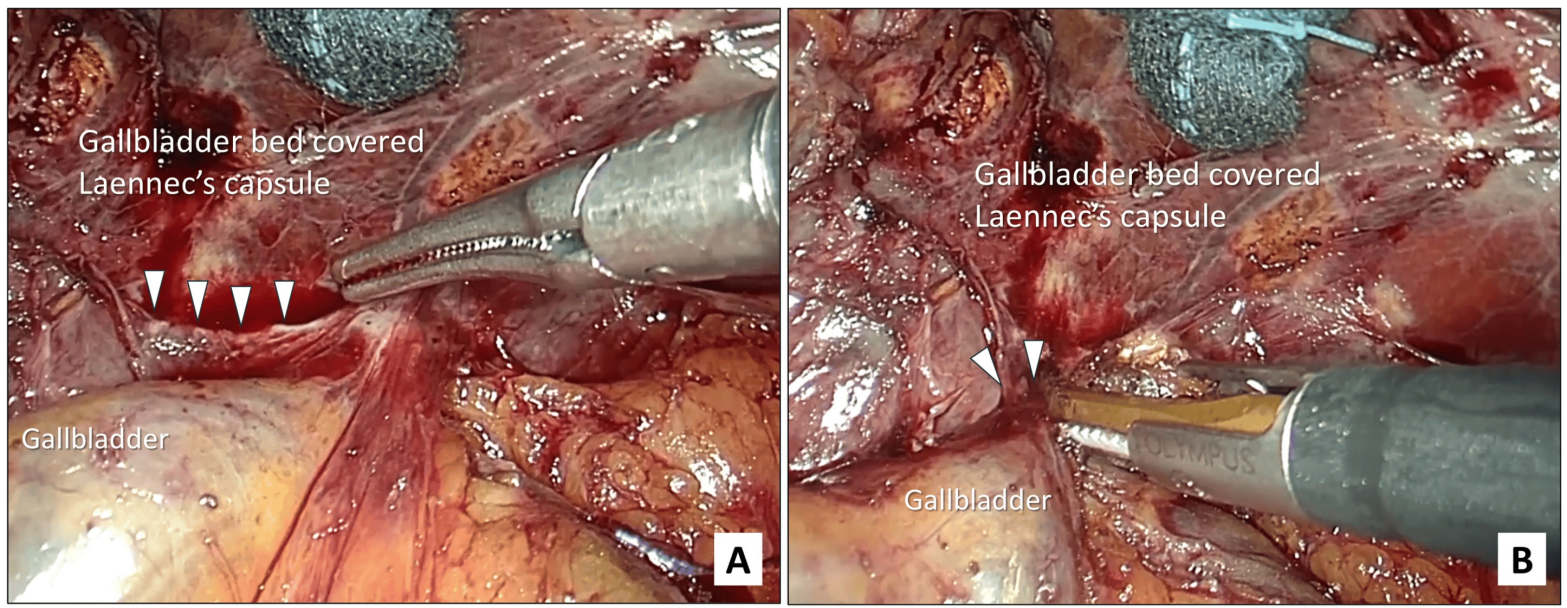
Intraoperative photographs showing visualization and transection of the cystic plate. A. The cystic plate (white arrowheads) is recognizable as a band-shaped structure on the neck. B. The cystic plate (white arrowheads) is transected with laparoscopic coagulating shears.

**Figure 5:**
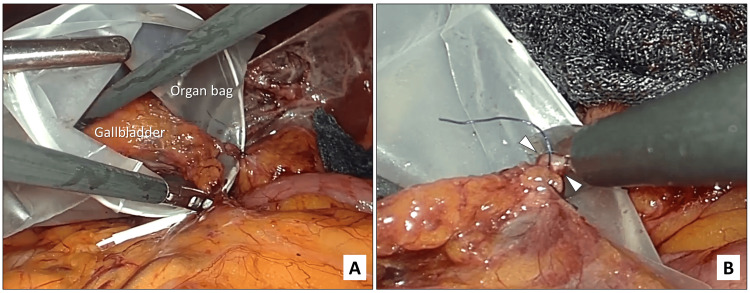
Intraoperative photographs showing resection of the cystic duct after the gallbladder was placed in an organ bag. A. Placement of the gallbladder, including the cystic duct, into an organ bag. B. The cystic duct (white arrowheads) is transected with laparoscopic scissors while completely contained within an organ bag.

(4) Visual identification and transection of the cystic plate: Dissecting the gallbladder from the liver bed using a fundus-first technique results in isolation of the cystic plate at the gallbladder neck (Figure 4A). After the cystic plate is reliably visualized, it is transected to complete the separation of the gallbladder from the liver bed (Figure 4B). With the transected cystic plate as a boundary, Laennec's capsule is preserved on the surface of the liver bed, and the hilar plate is preserved on the side of the hepato‑duodenal ligament.

(5) Resection of the cystic duct after placement of the gallbladder in an organ bag: In the final step, the gallbladder is placed in an organ bag after double ligation of the cystic duct (Figure 5A). The cystic duct is then transected with laparoscopic scissors while the cystic duct is securely held within the organ bag to avoid intraperitoneal dissemination of bile (Figure 5B). The bag containing the gallbladder is then removed via the umbilical incision, and the cystic duct stump is submitted for rapid intraoperative pathological examination. Once the gallbladder has been completely separated, the organ bag is extracted through the umbilical incision, after which the cystic duct stump is submitted for intraoperative frozen section diagnosis.

Pathological Assessment of the Dissected Surface of the Gallbladder

Achievement of “whole-layer” cholecystectomy is confirmed by pathological assessment. The surface of the gallbladder dissected from the liver bed is evaluated by examining an Elastica van Gieson (EVG) stained, formaldehyde-fixed specimen. In our institution, a certified pathologist evaluates paraffin-embedded tissue sections using NDP.view2 software (U12388-01; Hamamatsu Photonics, Shizuoka, Japan).

Postoperative Follow-Up of Patients With GBC

The GBC patients visited the outpatient clinic every three months after discharge. Enhanced computed tomography is performed at each visit to check for recurrence, and serum concentrations of tumor markers, such as carcinoembryonic antigen (CEA) and carbohydrate antigen 19-9 (CA19-9), are measured. The patients generally do not receive adjuvant chemotherapy during follow-up. 

Statistical Analysis

Variables that are not normally distributed are reported as median (range).

Results

The characteristics in the 15 patients with GBC, and the surgical and short-term results in the 14 patients with GBC who completed LWLC are shown in Tables 1-2, respectively.

**Table 1:** Patient characteristics of gallbladder cancer (GBC) cases. NR: normal range; BMI: body mass index; CEA: carcinoembryonic antigen; CA19-9: carbohydrate antigen 19-9; PET: positron emission tomography; SUV: standardized uptake value

Variables	Median (range) or numbers
Age (years)	70.5 (58-82)
Gender (male/female)	6/9
BMI (kg/m^2^)	23.0 (18.1-27.2)
Findings of cholecystitis (+/-)	4/11
CEA (ng/mlL; NR: 0-5.0)	2.1 (0.8-5.4)
CA19-9 (U/mL; NR: 0-37.0)	31.6 (9.0-181.3)
PET SUVmax	8.2 (2.1-24.1)
Tumor size (mm)	28.0 (19.9-43.8)

**Table 2:** Surgical and short-term results of the patients with GBC who completed LWLC. GBC: gallbladder cancer; GBI: gallbladder injury; BDI: bile duct injury; LWLC: laparoscopic whole-layer cholecystectomy

Variables	Median (range) or numbers
Operation time (min)	136 (96-176)
Blood loss (mL)	27 (0-104)
Blood transfusion	0
Intraoperative GBI or BDI	0
Postoperative complications	1
Postoperative hospital stay (days)	5 (3-14)

Cholecystitis was found in 26.7% of GBC patients. No patients underwent gallbladder drainage preoperatively. Regarding tumor markers, these were not outside the normal range on CEA and CA19-9 (median CEA, 2.1 ng/mL; median CA19-9, 31.6 U/mL). The median standardized uptake value max in PET was 8.2, and the median tumor diameter was 28.0 mm (range 19.9-43.8 mm). In the 14 patients with GBC who completed LWLC, the median operation time was 136 min (range 96-176 min), and the median estimated blood loss was 27 mL (range 0-104 mL). None of the patients required a blood transfusion during the operation. There were no instances of intraoperative gallbladder or bile duct injury. One patient had a postoperative abdominal abscess (Clavien-Dindo Grade II) that resolved with antibiotic treatment alone [13]. The median postoperative hospital stay was five days (range 3-14 days). There were no deaths. For information, of the 15 GBC patients, there was one patient converted to open surgery (No. 9 in Table 3). This patient was found by frozen section examination to have positive 12c lymph nodes and therefore underwent conversion to open surgery for resection of the extrahepatic bile duct with regional lymph nodes dissection. The oncological outcomes are summarized in Table 3.

**Table 3:** Oncological outcomes of GBC Gf: gallbladder fundus; Gb: gallbladder body; Gbf: gallbladder body and fundus; Gfb: gallbladder fundus and body; perit: peritoneal-side; ant: anterior-side; M: mucosa; MP: muscle propria; SS: subserosa; LN: lymph node; ly: lymphatic invasion; v: venous invasion; GBC: gallbladder cancer

No.	Tumor location	Type	T factor	LN	ly	v	Recurrence	Prognosis	Follow-up period (month)
1	Gf, perit	Others	Tis	N0	0	0	none	alive	143
2	Gf, perit	Nodular	T2, SS	N0	0	0	none	alive	76
3	Gbf, perit	Papillary	T1b, MP	N0	0	0	none	alive	68
4	Gb, perit	Flat	Tis	N0	0	0	none	alive	68
5	Gf, ant-perit	Papillary	T1a, M	N0	0	0	none	alive	67
6	Gf, perit-ant	Papillary	T1b, MP	N0	0	0	none	alive	65
7	Gf, perit	Others	Tis	N0	0	0	none	alive	56
8	Gfb, perit-ant	Nodular	T1b, MP	N0	0	0	none	alive	55
9	Gfb, perit	Nodular	T2, SS	N1	1	0	none	alive	53
10	Gf, perit	Papillary	T1a, M	N0	0	0	none	alive	41
11	Gf, perit	Nodular	T1a, M	N0	0	0	none	alive	36
12	Gf, perit	Flat	Tis	N0	0	0	none	dead	34
13	Gf, perit	Nodular	T1b, MP	N0	0	0	none	alive	31
14	Gb, perit	Nodular	T1a, M	N0	0	0	none	alive	26
15	Gf, Perit	Nodular	T1b, MP	N0	0	0	none	alive	17

All tumors were located on the peritoneal side. Regarding the T factor, the tumors were limited to the muscularis propria in 13 patients, whereas the subserosa was involved in two. Lymph node metastasis was detected in only one patient, the one who underwent conversion to open surgery. The median duration of follow-up of the 15 patients was 56 (17-143) months. No recurrences of GBC were identified during follow-up; however, one patient died of pulmonary disease.

**Figure 6:**
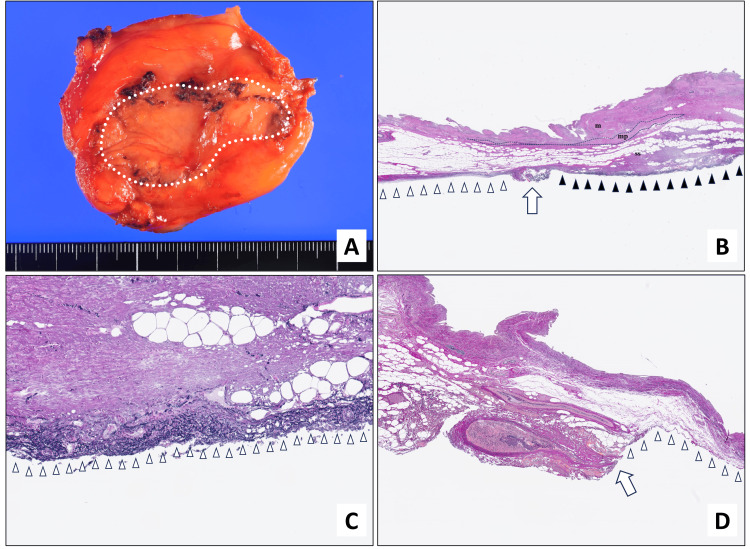
Macroscopic and microscopic findings of the dissected surface from the liver bed of the gallbladder. A. Photograph of operative specimen showing serosal and dissected surfaces of the liver bed (area encircled with white dotted line). B. Photomicrograph of an EVG-stained section of the gallbladder. White arrowheads: serosal surface; white arrow: first point of transection in whole-layer dissection of the gallbladder; black arrowheads: surface of the liver bed after dissection; m: mucosa; mp: muscularis propria; ss: subserosa. C. Photomicrograph of EVG-stained section of the gallbladder (white arrowheads: the surface of the liver bed after dissection). D. Photomicrograph of EVG-stained section of the gallbladder neck (white arrowheads: stump of cystic plate; white arrow: subserosal surface). EVG: Elastica van Gieson

Finally, we confirmed that “whole-layer” cholecystectomy was completely successful in all patients by pathological assessment. Macroscopically, after being dissected from the surface of the liver bed, the gallbladder was distinguished by having a uniform, glossy appearance (Figure 6A). The wall of the gallbladder has three layers: the mucosa, muscularis propria, and subserosa. It differs from the gastrointestinal tract in that it lacks a muscularis mucosa and submucosa. EVG staining revealed the presence of elastic fibers covering the outer layer of subserosa at the surface of the liver bed after dissection (Figures 6B, 6C). Furthermore, the stump of a bundle of elastic fibers, which we consider to be the transected cystic plate, was visible at the gallbladder neck (Figure 6D).

## Discussion

It has been reported that 37% to 74% of patients with early-stage GBC, defined as only invading the muscularis propria layer, are diagnosed preoperatively [10,14]. In our study, 34.1% of patients who had undergone LWLC with a preoperative diagnosis of suspected GBC were found by pathological examination of the operative specimen to indeed have GBC. In contrast, less than 10% of gallbladder polyps larger than 10 mm prove to be invasive cancers [15]. Thus, it is difficult to make a definitive preoperative diagnosis of early-stage GBC. Patients with findings suspicious of GBC should be handled as if they have GBC and undergo a surgical procedure that offers both oncological safety and minimal invasiveness. Furthermore, it is essential to establish techniques for laparoscopic surgery that successfully address intraoperative concerns such as gallbladder perforation and incomplete resection of the gallbladder.

In this study, we present our original surgical technique and the steps in this procedure for suspected early GBC. Recently, favorable surgical outcomes have been reported for laparoscopic cholecystectomy and LWLC for definite or suspected early GBC [16,17]. However, to the best of our knowledge, the operative procedures have not been reported in detail. Our article differs in this respect from previous reports. We believe that our description of our original surgical procedure and the steps required to perform it successfully will be of great help in overcoming the drawbacks of laparoscopic surgery for suspected early GBC.

An important focus of our LWLC is thorough prevention of bile leakage, which necessitates avoiding grasping the gallbladder as much as possible. This can be facilitated by demarcating the surgical field using folded gauze. In addition, when grasping the gallbladder, it is important to use fine-tipped forceps, such as precision forceps, and to grasp the gallbladder serosa delicately with the tips of the forceps. Another possible source of bile leakage is transection of the cystic duct. The risk of this can be minimized by transecting the cystic duct after the gallbladder, including the cystic duct, is placed in an organ bag. With the implementation of these maneuvers, none of our patients had intraoperative bile leakage.

With respect to lymph node dissection, we limited ourselves to sampling of the 12c lymph nodes. It has recently been reported that the rate of lymph node metastasis from GBCs that are invading only the muscularis propria layer ranges from 0% to 14.8% [18,19]. Vo et al. found lymph node metastases in 32 (14.8%) of 217 patients with GBCs invading the muscularis propria layer and concluded that lymph node dissection was necessary because lymph node dissection and postoperative adjuvant chemotherapy contributed to an improved survival rate [18]. In contrast, de Aretxabala et al. investigated 49 patients with GBCs invading the muscularis propria layer and found no instances of lymph node metastasis [19]. It should be noted that the reliability of the diagnosis of depth of invasion by de Aretxabala et al. would have been extremely high because they evaluated the entire tumor pathologically [19]. On the basis of the above findings, we consider it reasonable to limit lymph node dissection to sampling of the 12c lymph nodes when performing LWLC for suspected early GBC. Extensive lymph node dissection within the hepatoduodenal ligament carries a risk of common bile duct injury and postoperative bile duct stenosis due to ischemia. Regardless of whether the 12c lymph node can serve as a sentinel lymph node, we found no metastases to 12c lymph nodes in our series of patients with GBC that only invaded the muscularis propria layer.

The most important consideration when performing LWLC is the establishment of the process checkpoints and anatomical landmarks, with the process checkpoints specifically defined as the five steps of LWLC. Reviewing each step on its completion enables smooth accomplishment of LWLC. The hilar plate [7], cystic plate, and Laennec’s capsule [8] are particularly important anatomical landmarks. In the first step, the cystic artery and duct are isolated, and the hilar plate is preserved. In the third step, the gallbladder is dissected from the liver bed, with the gallbladder side covered by the cystic plate, from the fundus to the neck of the gallbladder, enabling recognition of the cystic plate as a band-shaped structure at the neck. The entire gallbladder is resected, and the right anterior Glissonean pedicle is reliably preserved by using the isolated cystic plate as a landmark for the boundary of the dissection layer between the neck and body. Laennec’s capsule, a fibrous capsule, is peculiar to the liver [20]. Preservation of Laennec’s capsule on the liver side of the liver bed prevents exposure of the liver parenchyma during LWLC. This enables avoidance of the difficult-to-control bleeding that can result from injury to the branches of Glisson and/or the middle hepatic vein. This explains why preservation of Laennec’s capsule on the liver bed is key to this minimally invasive procedure. When Laennec’s capsule is preserved, relatively large branches of the middle hepatic vein can be seen through it.

Finally, we confirmed that the gallbladder had been resected intact and with clear margins by pathological evaluation of the liver bed side of the gallbladder. Pathological assessment of the resected specimen revealed that exposure of the subserosa was prevented by the presence of elastic fibers stained with EVG. The glossy and uniform dissected surface of the liver bed, seen macroscopically, is in accordance with the membranous structure of elastic fibers shown by EVG staining. To the best of our knowledge, Honda et al. were the first to pathologically demonstrate the presence of these elastic fibers [21]. Our findings on pathological examination of the dissected surface of the liver bed are consistent with those presented in their report, supporting our contention that our procedure enables the accomplishment of complete, intact laparoscopic cholecystectomy. Honda et al. also speculated that Laennec’s capsule and the visceral peritoneum or peritoneal remnant tissue of the gallbladder overlap in the liver bed, and that the layer between these membranous structures can be separated. Our findings on pathological assessment indicate that, in the third step of our surgical procedure, it is possible to dissect the gallbladder from the liver bed while maintaining a glossy layer on both the liver and gallbladder sides. Furthermore, it is possible to reliably resect the gallbladder complete with a membranous covering, enabling prevention of uncontrollable bleeding from the liver bed.

## Conclusions

In conclusion, we have demonstrated that our five-step version of LWLC with its maneuvers for preventing perforation of the gallbladder is a feasible, minimally invasive, and reproducible surgical procedure that can achieve oncological cure of definite and suspected early GBC.
